# Cellular effects of oral egg yolk immunoglobulin-based supplementation at birth on promoting growth and strengthening intestinal mucosal innate immunity in pre-weaned piglets

**DOI:** 10.3389/fvets.2025.1458279

**Published:** 2025-07-16

**Authors:** Muttarin Lothong, Autchara Kayan, Manmueang Malison, Dran Rakarcheep, Theerawat Samritwatchasai, Sumpun Thammacharoen, Chatsri Deachapunya, Sutthasinee Poonyachoti

**Affiliations:** ^1^Department of Animal Science, Faculty of Agriculture, Kasetsart University, Bangkok, Thailand; ^2^Department of Physiology, Faculty of Veterinary Science, Chulalongkorn University, Bangkok, Thailand; ^3^Department of Physiology, Faculty of Medicine, Srinakharinwirot University, Bangkok, Thailand; ^4^Center of Excellence in Animal Fertility Chulalongkorn University (CU-AF), Chulalongkorn University, Bangkok, Thailand

**Keywords:** immunoglobulin Y, growth, mucosal innate immunity, low birth weight, pre-weaned piglets, intestinal barrier

## Abstract

Low birth weight harms growth and immunity in suckling piglets. The effects of chicken egg yolk immunoglobulin (IgY)-based product, Globigen^®^ Pig Doser (EW Nutrition GmbH, Visbek, Germany) (GPD), administered at birth on growth performance at weaning were investigated in normal (NBW) and low birth weight (LBW) piglets. The product comprises IgY antibodies against common enteric pathogens in newborn piglets, such as *Escherichia* spp., antibodies against common enteric pathogens in newborn piglets, such as *Escherichia* spp., as well as soybean oil, vitamins, and probiotics like *Enterococcus faecium*. The expression of mRNA and protein related to mucosal innate immunity was measured using real-time quantitative polymerase chain reaction (qPCR) and Western blot in 7-day-old piglets (*n* = 5 piglets). LBW (0.8–1.0 kg birth weight [BW]) and NBW (1.4–1.6 kg BW) piglets were randomly chosen from different sows. At 6 and 10 h after birth, 1.5 mg of IgY (NBW-IgY or LBW-IgY piglets; *n* = 32 piglets/group) was orally supplemented. GPD significantly increased the final BW and daily weight gain of NBW-IgY but not LBW-IgY piglets at 24 days after birth compared to the untreated group. The growth performance of LBW-IgY piglets was improved to match NBW piglets. The expressions of antimicrobial peptides, porcine *β***-**defensin *(PBD)4*, pathogen recognition receptors, toll-like receptor *(TLR)1*, *TLR2*, *TLR5*, *TLR6*, *TLR8*, and *TLR9*, and cytokines *IL-6* were enhanced in NBW-IgY. The upregulated expressions of *PBD*2 and *PBD4* were observed in the jejunal mucosa of LBW-IgY piglets. GPD reversed the overexpression of neuropeptides substance P, calcitonin gene-related peptides, and cytokines interleukin 8 (*IL-8*) that underlie inflammation in the LBW group. The LBW group exhibited elevated expression of tight junction (TJ) barrier proteins, including claudin (CLDN)4, CLDN7, and ZO-1 in the colon, but these levels were reversed by GPD. Our findings indicated that oral GPD supplementation at birth can promote growth by modulating the intestinal barrier system and reducing the incidence of inflammation in pre-weanling piglets.

## 1 Introduction

The larger litter size of hyperprolific sows is linked to a higher number of low birth weight (LBW) piglets. The variation in body weight of suckling piglets causes management difficulties, such as an irregular start to the finishing period. LBW piglets are linked to pre-weaning mortality, growth retardation, and reduced carcass quality (1). According to Morise et al., LBW piglets weigh 0.8–1 kg, while normal birth weight (NBW) piglets weigh between 1.4 and 1.6 kg (2). Piglets of the LBW group are more vulnerable to pathogens, which can cause long-term negative effects on growth performance during the farrowing and finishing phases (3).

Intestinal epithelial cells (IECs) are crucial for piglet health, including their roles in digestion, absorption, and immunity (4). Intestinal immune systems involve a complex interaction between innate and adaptive components to defend against pathogens, while also tolerating commensal microbes and dietary antigens. The multilayered system of chemical and physical barrier functions, consisting of intestinal epithelium, immune system, and enteric nervous system, serves as the first line of the innate immune response, working to prevent invading pathogens. The most effective chemical barrier is antimicrobial peptides, such as *β*-defensins (BDs) secreted by IECs and immune cells (5), while the physical barrier is maintained by tight junction (TJ) protein complexes (zona occludin and claudins) (6, 7).

When the host–microbe interaction occurs, IECs initiate the synthesis of proinflammatory cytokines (interleukin (IL)-1β, IL-6, IL-8, and tumor necrosis factor (TNF)-*α*). This represents the second line of the innate immune system, which involves pattern recognition receptors (PRRs), toll-like receptors (TLRs; TLR1-10), and the signaling system expressed by IECs or macrophages and dendritic cells beneath the IECs (7). The recognition of pathogens by PRRs activates intracellular signaling pathways that influence subsequent immune responses, such as the activation of B and T lymphocytes, which provide long-lasting protection against specific pathogens (8). Moreover, regulatory T lymphocytes secrete inhibitory cytokines (transforming growth factor-*β* (TGF-β) and IL-10) to prevent overactive immune responses to harmless antigens and to maintain immune tolerance to commensal bacteria and dietary antigens (9).

Various factors, including diet, microbial exposure, and genetic background, influence the development of the intestinal immune system in piglets. Thus, LBW piglets, which may be caused by an inadequate intrauterine nutrients or growth factors, might experience improper GIT development. Moreover, due to the epitheliochorial type of pig placenta, maternal immunity prevents the transfer of antibodies to the fetuses (10). Therefore, maternal immunity through colostrum and milk is the only protection against disease (3).

Maternal colostrum containing antibodies and immune cells is only transferred from the sow to the piglets across the intestinal wall, which is highly effective in the first 24 h after birth (10). Therefore, ingesting colostrum shortly after birth is critical for newborn piglets to receive passive immunity. Nevertheless, the colostrum supply is often insufficient for the number of piglets that exceeds the number of functional teats of the sow, especially for the last-born piglets and the LBW piglets (11).

Animals can receive passive immunity from colostrum, egg yolks, and monoclonal antibodies from various sources (12). Feeding bovine colostrum to piglets has been reported (13). Although bovine colostrum is rich in nutrients and bioactive compounds, the immunoglobulins in bovine colostrum target bovine pathogens (14) and are probably unable to prevent pig-specific infections. Perhaps, passive immunization can enhance the innate immune response to infection, resulting in improved weight gain during the weaning period, especially in LBW piglets.

Immunoglobulin Y (IgY) is a low molecular weight class of immunoglobulin (Ig) present in avian serum and egg yolk of birds, amphibians, and reptiles (15). Similar to mammal IgG, IgY is responsible for the secondary immune response (16). The IgY-based commercial product, Globigen^®^ Pig Doser (EW Nutrition GmbH, Visbek, Germany; GPD), contains high-purity IgY against antigens causing diarrhea, such as enterotoxigenic *Escherichia coli* (ETEC) F4 and F18, *E. coli* K88, and *Salmonella typhimurium* (17). Additionally, preventing the colonization of ETEC *E. coli* K88 by oral IgY in newly weaned piglets at 10 days of age showed an improvement in weight gain (18). Therefore, IgY-based commercial products are widely used in many applications, including prophylactics, therapeutics, and functional foods to promote growth (19). However, the cellular mechanisms of IgY-based supplementation only at birth on the intestinal barrier system, which may be important targets for improving gut health associated with growth performance, have not been investigated.

In the current study, we aimed to investigate whether oral IgY-based supplementation within 24 h after birth promotes the expression of innate immune system-related genes in connection with growth in weaning piglets. Comparisons between LBW and NBW piglets were also conducted to develop strategies for reducing economic losses from managing or culling weak post-weaned piglets.

## 2 Materials and methods

### 2.1 Chemicals

The commercially produced IgY, Globigen^®^ Pig Doser, was provided by EW Nutrition GmbH (Visbek, Germany). Radioimmunoprecipitation assay (RIPA) reagent and a protease inhibitor cocktail were purchased from Merck Group (Darmstadt, Germany). Reagents used for Western blotting were purchased from Bio-Rad, Inc. (Hercules, CA, United States). The body composition analysis (BCA) test and enhanced chemiluminescence (ECL) substrate were purchased from BCA Test Visual Protein (Taipei, Taiwan). All primary antibodies, including CLDN1, rabbit anti-CLDN2, rabbit anti-CLDN4, rabbit anti-CLDN7, and mouse anti-β-actin antibody, were purchased from Santa Cruz Biotechnology (Dallas, TX, United States), and secondary antibodies from Cell Signaling Technology (Danvers, MA, United States). RNAlater™ solutions and TRIzol^®^ reagent were purchased from Thermo Fisher Scientific (Waltham, MA, United States). iScript™ Select cDNA Synthesis Kit was purchased from Bio-Rad, Inc. (Hercules, CA, United States). The 2X qPCRBIO SyGreen Mix Lo-ROX was purchased from PCR Biosystems Ltd. (N6 4ER, London, United Kingdom). The specific primer sets were designed and developed by iScience Technology (Bangkok, Thailand).

### 2.2 Animals and experimental procedures

The animal experiment was conducted in accordance with the animal use protocols approved by the Faculty of Veterinary Science, Chulalongkorn University Animal Care and Use Committee (protocol number: 2231047). The basal diets used to meet the requirements for suckling piglets according to the Veterinary Manual: MSD Veterinary Manual (20) as discussed in the following.

A total of 128 piglets [(Landrace × Yorkshire) × Duroc] from 16 sows of the same parity (parity 2) were randomly assigned to four experimental groups (*n* = 32 piglets/group). The sample size was determined through power analysis (G*Power software (version 3.1.9.2; Heinrich Heine University Düsseldorf, Düsseldorf, Germany)) for a two-factor interaction. The input parameters include a medium effect size (Cohen’s d = 0.25) with a significance level of 0.05, a power of 0.8, and a numerator *df* of 1 to determine the sample size for 4 treatments. The nursing sows and suckling piglets were housed in temperature- and humidity-controlled environments using an evaporative cooling system. During the experiment, the barn maintained a daily temperature of 26.5 ± 0.9°C and relative humidity of 71.0 ± 1.0%. The piglets were separated from their mothers and weighed prior to receiving colostrum. The piglets with a birth weight of 1.4–1.6 kg were assigned to the NBW group, and those with a birth weight of 0.8–1.0 kg were assigned to the LBW piglets. After group allocation, all piglets were returned to their mothers and fed with the sow’s colostrum. Some NBW and LBW piglets (NBW-IgY and LBW-IgY) were orally supplemented with 2 mL of GPD, which is is equivalent to orally pump-fed-IgY (0.3 mg of IgY/ml) (21), administered at 6 and 10 h after birth to simulate the receiving of passive immunity from colostrum. The piglets were cross-fostered according to the farm’s standard practices. Each sow was allotted 13–14 piglets/litter. At 7 days after birth, piglets were cross-fostered again and were further weaned when they were 24 days old. During cross-fostering, the number of piglets per sow per litter and the size of the piglets within each litter will be monitored to ensure that they are similar. Creep feed (with 2.58 Mcal/kg, 20.8% crude protein, 2.5% crude fiber, 0.60% calcium, and 0.45% available phosphorus) and water were provided *ad libitum* throughout the entire 24-day experimental period, following the farm’s protocol. The piglets were weighed on a pen basis on days 0, 7, 8, and 24 to calculate the average daily gain (ADG).

### 2.3 Sample collection

Seven-day-old piglets from each experimental group (*n* = 5 piglets per group) were randomly selected and euthanized to remove the gastrointestinal tract. Immediately after collection, a 1 cm length of intestinal mucosa from the mid-jejunum, ileum, and colon was placed into a 2 mL microcentrifuge tube containing RNAlater and snap-frozen in liquid nitrogen. All mucosal samples were kept at −20°C for further analysis.

### 2.4 RNA isolation and cDNA synthesis

Total RNA was extracted using TRIzol^®^ reagent following the manufacturer’s protocol. Briefly, 1 g of frozen mucosal sample was homogenized on ice in 1 mL of TRIzol^®^ using a homogenizer pestle. After 200 μl of chloroform was added, the mixture was centrifuged at 12,000 *g* for 15 min. The upper layer containing RNA was transferred into a new microfuge tube and precipitated with 95% isopropanol. The RNA pellet was rinsed with 70% ethanol and air-dried at room temperature. After reconstitution with nuclease-free water, RNA samples with a ratio between 1.8 and 2.0 at a wavelength of 260/280 nm, as determined by NanoDrop spectrophotometer (Thermo Scientific, Waltham, MA, USA), were used for the next step.

Complementary DNA (cDNA) was synthesized with iScript™ Select cDNA Synthesis Kit according to the product’s instructions. One microgram of total RNA template was mixed with 5 × reaction mixture, which included 4 μL oligo-(dT)20 primer, 1 μL iScript reverse transcriptase, and nuclease-free water adjusted to 20 μL. The reactions were performed in a thermocycler (Bio-Rad, Inc., Hercules, CA, United States) following steps: denaturation at 65°C for 5 min, primer hybridization at 42°C for 30 min, reverse transcription at 25°C for 5 min, and enzyme inactivation at 85°C for 5 min. The cDNA product was stored at −20°C for the next step.

### 2.5 Real-time polymerase chain reaction

The mRNA expression of mucosal immunity-related genes, tight junction proteins, and neuropeptides was determined using real-time PCR with a CFX96 Real-Time PCR Detection System (Bio-Rad Laboratories, Hercules, CA, USA) and the 2X (PCR Biosystems Ltd., London, United Kingdom) Lo-ROX as previously described (22). The specific primer sets listed in Table 1, which followed those previously studied (22), were used. The expression of certain genes was normalized as a fold change (*2*^−*ΔCt*^; *Ct* = the threshold cycle) relative to the reference gene (glyceraldehyde-3-phosphate dehydrogenase; *GAPDH*). Following the equation of the *2*^−*ΔΔCt*^ method (23), semiquantitative of gene expression in all groups was calculated as fold changes from the mean of the NBW group. The amplicons were checked for product specificity by running 1.5% agarose gel electrophoresis and analyzing the melting curve.

**Table 1:** Sequences of specific primer sets used for real-time quantitative PCR.

Gene	Nucleotide sequences (5′-3′)	Accession number
*PBD1*	F: TGCCACAGGTGCCGATCT R: CTGTTAGCTGCTTAAGGAATAAAGGC	NM-213838.1
*PBD2*	F: CCAGAGGTCCGACCACTACA R: GGTCCCTTCAATCCTGTTGAA	NM_214442.2
*PBD3*	F: CCTGATGCCTCTTCCAGGTAAT R: TTTCGGCCACTCACAGAACA	NM_214444.1
*PBD4*	F: GGGTCACAATTTTACCAGCCAG R: CATCGACCATTGCCCCTTCT	NM_214443.1
*CLDN1*	F: CCCGGTCAATGCCAGATATG R: CACCTCCCAGAAGGCAGAGA	NM_001161635.1
*CLDN2*	F: CTCCCTGATAGCTGGGATCATC R: CCTGATAGGCATCGTAGTAGTTGGA	NM_001161638.1
*CLDN4*	F: GTGTAAGGTGCTACCGCTGATTC R: AGGGCCATTCTGGAGTCACA	NM_001161637.1
*CLDN5*	F: ACCGGCGACTACGACAAGAA R: GCCCTCCAAAGCGGAGTT	NM_001161636.1
*CLDN7*	F: CCATGACTGGAGGCATCATTT R: GACAATCTGGTGGCCATACCA	NM_001160076.1
*ZO-1*	F: CTTGACCCTAGACAGCACCCTGA R: GTGCATCATAGGCAGGAGTGGA	XM_005659811.1
*TLR1*	F: CACAGAGTCTGCACATTGTTTATCC R: GATTTACTGCGGTGCTGACTGA	NM_001031775.1
*TLR2*	F: GTGCTTTCCGAGAACTTTGT R: GCAGAATGAGGATGGCG	KF460452.1
*TLR3*	F: TCCAACTAACAAACCAGGC R: ACATCCTTCCACCATCT	NM_001097444.1
*TLR4*	F: AAGGTTATTGTCGTGGTGT R: CTGCTGAGAAGGCGATAC	NM_001293316.1
*TLR5*	F: TTGCATCCAGATGCTTTTCA R: TTCAACTTCCCAAATGAAGGA	XM_012506471.1
*TLR6*	F: TCACCTCTCTGACATCAGCTTTCT R: TGATATCAAGGCACTGCATCCT	NM_213660.1
*TLR7*	F: GGACCATCTGGTAGAGATCGATTT R: TTCTGGTGCACAGGTTGTCTTT	NM_001097434.1
*TLR8*	F: CCGCACTTCGCTATCTAAAC R: GAAAGCAGCGTCATCATCAA	NM_214187.1
*TLR9*	F: AGATGTTTGCTCGCCT R: GGACACTCGGCTATGGA	KC860785.1
*TLR10*	F: CTACCAGGTATCCTGCACTGAAAG R: GGCAACATTTACGCCTATCCTT	NM_001030534.1
*TNF-α*	F: ATCGGCCCCCAGAAGGAAGAG R: GATGGCAGAGAGGAGGTTGAC	M29079 X54859
*IL-6*	F: AACGTGCAGTCTATGGAGT R: GAACACCACTTCTCTCTTCA	NM_214399
*IL-8*	F: TTTCTGCAGCTCCTCTGTGAGG R: CTGCTGTTGTTGTTGCTTCTC	M99367 M86923
*IL-10*	F: CTGCATCCACTTCCCAACCA R: TGGCAACCCAGGTAACCCTTA	NM_214041.1
*IFN-γ*	F: GTTTTTCTGGCTCTTACTGC R: CCTCCGCTTTCTTAGGTTAG	X53085
*CALCB*	F: ATGGGCTTCGGGAAATCCTC R: CTCCAAAGCGGACCTGAGTG	NM_001104955.1 NM_001102473.1
*TAC1*	F: GTGGCCTTGGCAGTCTTTTTTC R: CTCCTTTATCTGGTCGCTGTCTG	XM_003130164.6 XM_013979715.2
*GAPDH*	F: GGACCAGGTTGTGTCCTGTGA R: TCCACCACCCTGTTGCTGTAG	NM_001206359.1

### 2.6 Semi-quantitative Western blot analysis

The mucosal samples were lysed with RIPA buffer containing 50 mM tris, 150 mM NaCl, 1 mM ethylene glycol-bis(*β*-aminoethyl ether)-N, N, N′, N′-tetraacetic acid (EGTA), 1 mM phenylmethylsulfonyl fluoride (PMSF), 1% NP-40, 6.02 mM sodium deoxycholate, 0.01 mg/mL aprotinin, 1 mM NaF, and a cocktail protease inhibitor. After centrifugation, the supernatant was collected, and its protein concentration was measured using the BCA™ protein assay (Thermo Fisher Scientific, MA, United States). The lysate sample was incubated with Laemmli buffer containing *β*-mercaptoethanol (Bio-Rad, Inc., Hercules, CA, United States) at 65°C for 5 min. The denatured protein (30 μg) was separated using sodium dodecyl sulfate-polyacrylamide gel electrophoresis (SDS-PAGE) and transferred onto a polyvinylidene fluoride microporous membrane (Millipore^®^, St. Louis, MO, United States). After blocking the non-specific binding proteins with 5% non-fat dried milk, the blotted membrane was incubated overnight with primary antibodies, followed by secondary antibodies. The dilution of antibodies was prepared according to the manufacturer’s recommendations. After being developed with an ECL substrate (Santacruz Biotechnology, Dallas, TX, United States), the immunoreactive band was detected by gel documentation. The band density was analyzed by densitometric Scion Image Software 4.0.3.2 (NIH, Bethesda, MD, United States). The ratio of the target protein band intensity was compared with that of β-actin. All experiments were performed in duplicate to ensure reproducibility.

### 2.7 Statistical analysis

All data were expressed as mean ± standard error of the mean (SEM) of 4 independent experiments from 5 to 32 piglets per group. A comparison of different treatments on varying birth weights was performed using two-way analysis of variance (ANOVA) followed by Tukey’s *post hoc* test. One-way ANOVA was used to evaluate gene expression, followed by Tukey’s multiple comparison test. The statistical analysis was done using GraphPad Prism 9.0 (GraphPad Software, Inc., CA, USA). A significant difference was considered at a 95% confidence interval (CI; *p* < 0.05).

## 3 Results

### 3.1 Effect of oral GPD on the growth performance of suckling piglets

The growth performance of piglets orally administered GPD was demonstrated in two distinct periods: 0–7 and 8–24 days. The average birth weight of NBW piglets was 1.48 ± 0.06, which was significantly higher than LBW piglets (0.95 ± 0.06; *p* < 0.05; Table 2). During the first week (days 0–7), both NBW and LBW piglets treated with GPD showed significant increases in ADG, despite no difference in the final birth weight (BW) at day 7 compared with the untreated group (*p* < 0.05; Table 2). During days 8–24, significant increases in both final weight and ADG were also observed in NBW-IgY piglets receiving oral GPD supplementation in relation to NBW piglets (*p* < 0.05; Table 2). No significant changes in the final weight and ADG were detected between GPD-treated LBW (LBW-IgY) and untreated LBW (Table 2) piglets. Moreover, the final weight of LBW-IgY piglets at 24 days after birth was not different from untreated NBW piglets (Table 2).

**Table 2:** Effect of oral GPD on growth performance of suckling piglets.

The average growth performance at each age duration	NBW piglets		LBW piglets		*p*-value		
	NBW piglets	NBW-IgY piglets	LBW piglets	LBW-IgY piglets	IgY	BW	IgY*BW
0–7 days							
Initial weight (kg)	1.48 ± 0.06^A2^	1.48 ± 0.05^A2^	0.95 ± 0.06^B2^	0.95 ± 0.05^B2^	0.507	<0.001	0.652
Final weight (kg)	2.55 ± 0.30^A1A2^	2.75 ± 0.33^B1A2^	1.99 ± 0.34^A1B2^	2.15 ± 0.31^B1B2^	0.012	<0.001	0.718
ADG (g/day)	161.2 ± 5.09 ^A1A2^	181.42 ± 4.09 ^B1A2^	148.57 ± 4.33^A1B2^	171.42 ± 3.75^B1B2^	0.004	<0.001	0.402
8–24 days							
Initial weight (kg)	2.53 ± 0.32^A2^	2.70 ± 0.40^A2^	2.02 ± 0.33^B2^	2.06 ± 0.32^B2^	0.209	<0.001	0.466
Final weight (kg)	5.84 ± 0.14^a^	7.00 ± 0.76^b^	4.95 ± 0.19^c^	5.40 ± 0.80^ac^	0.005	<0.001	0.001
ADG (g/ day)	194.71 ± 7.89^a^	252.94 ± 9.93^b^	170.58 ± 10.89^c^	196.47 ± 11.97^ac^	0.011	0.014	0.001
0–24 days							
ADG (g/day)	174.4 ± 4.43^a^	220.8 ± 7.80^b^	162.78 ± 7.22^c^	178.73 ± 9.35^ac^	0.003	<0.001	<0.001

ADG = average daily gain; NBW = normal birth weight; LBW = low birth weight. Distinct upper case letters indicate significant differences at *p* < 0.05 in main effects: A1 and B1 denote a significant difference between GPD treatment (IgY), as A2 and B2 show a significant difference between normal and low birth weight groups (BW). In the group exhibiting the main effect interaction (IgY*BW), only lower case letters were displayed. Different lower case letters indicate an interaction by two-way ANOVA followed by Tukey’s post hoc test.

### 3.2 Effect of oral GPD on the mRNA expression of porcine *β*-defensins in the intestinal mucosa of suckling piglets

**Figure 1:**
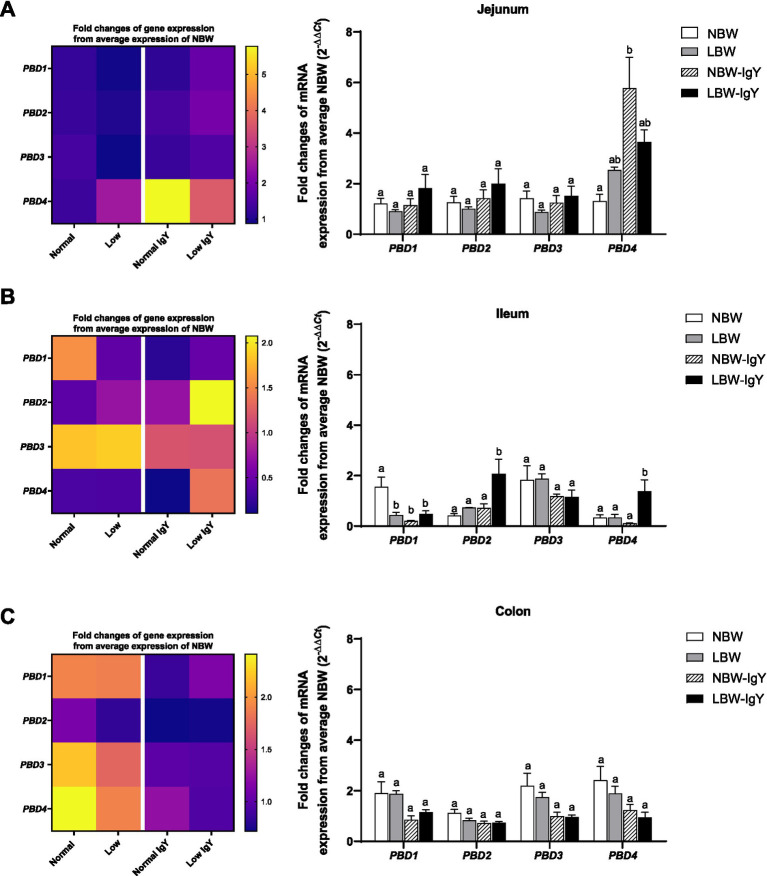
Effect of oral GPD supplementation on the porcine β-defensins (*PBD*) mRNA expression in **(A)** jejunal, **(B)** ileal, and **(C)** colonic mucosa of suckling piglets with normal birth weight (NBW) piglets vs. low birth weight (LBW) piglets. GPD was orally administered to NBW (NBW-IgY) and LBW (LBW-IgY) piglets at 6 and 10 h after birth. Total RNA was extracted from the intestinal mucosa of 7-day-old piglets. Expressions of *PBD1*, *PBD2*, *PBD3*, and *PBD4* were determined by real-time RT-PCR as relative fold changes from the mean of NBW piglets using 2*^−∆∆Ct^* calculation. The heat map represents the mean, and the bar represents the mean ± SEM of individual gene expression conducted in duplicated run (*n* = 5 piglets). The colors ranging from purple to yellow in the heat map for each gene reflect the fold changes in gene expression from lower to higher than in NBW piglets. Bar graphs with different letters (a or b) indicate significant differences among groups at *p* < 0.05 by one-way *ANOVA* followed by Tukey’s *post hoc* test.

In untreated NBW and LBW piglets, the mRNA expression of PBD1-4 in the intestinal mucosa was detected at a comparable level (1-fold change from NBW piglets; Figure 1), except that *PBD1* in the ileal mucosa of LBW piglets was lower than that of NBW piglets (*p* < 0.05; Figure 1A).

*PBD1-4* expression of NBW-IgY piglets did not differ from that of NBW piglets except that *PBD4* was upregulated (>6-fold *p* < 0.05; Figure 1A) in the jejunal mucosa, and *PBD1* was downregulated in the ileal mucosa (2-fold; *p* < 0.05; Figure 1B).

LBW-IgY piglets showed the highest levels of *PBD2* and *PBD4* expression in the ileal mucosa among all groups (*p* < 0.05; Figure 1A). Nevertheless, GPD treatment could not reverse the low-level expression of *PBD1* in the ileal mucosa of LBW piglets to the same level as NBW piglets (Figure 1B). Notably, no significant changes in all PBD expressions were detected in the colonic mucosa of untreated or GPD-treated NBW and LBW piglets (Figure 1C).

### 3.3 Effect of oral GPD on the mRNA expression of toll-like receptors in the intestinal mucosa of suckling piglets

**Figure 2:**
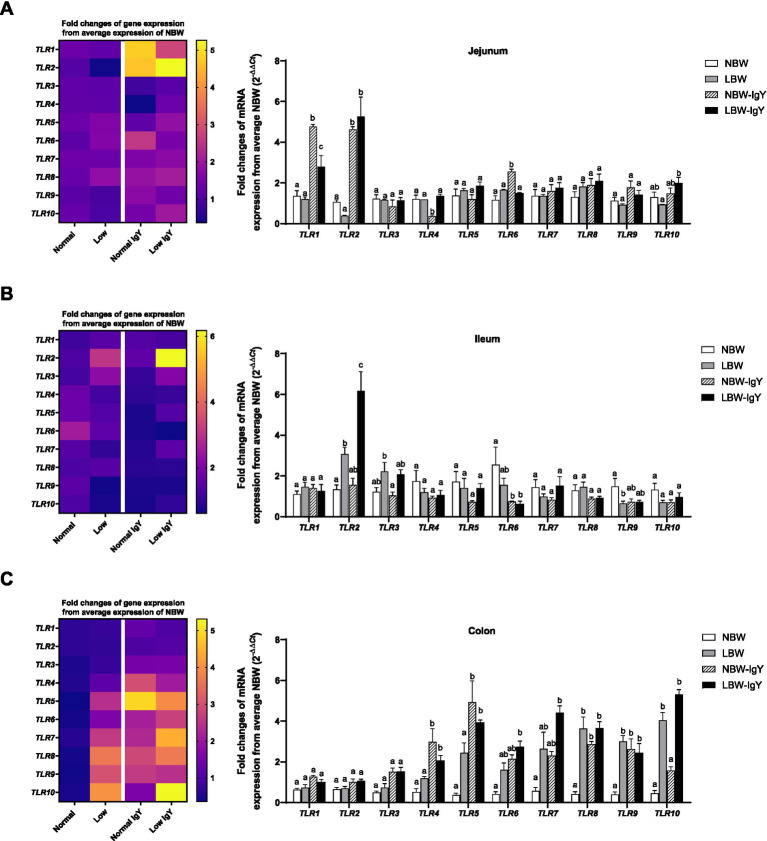
Effect of oral GPD on the toll-like receptors (*TLR*s) mRNA expression in **(A)** jejunal, **(B)** ileal, and **(C)** colonic mucosa of suckling piglets with normal birth weight (NBW) piglets vs. low birth weight (LBW) piglets. GPD was orally administered to NBW (NBW-IgY) and LBW (LBW-IgY) piglets at 6 and 10 h after birth. Total RNA was extracted from the intestinal mucosa of 7-day-old piglets. Expressions of *TLRs* (*TLR1* to *TLR10*) were determined by real-time RT-PCR as relative fold changes from the mean of NBW piglets using 2*^−∆∆Ct^* calculation. The heat map represents the mean, and the bar represents the mean ± SEM of individual gene expression conducted in duplicated run (*n* = 5 piglets). The colors ranging from purple to yellow in the heat map for each gene reflect the fold changes in gene expression from lower to higher than NBW piglets. Bar graphs with different letters (a or b) indicate significant differences among groups at *p* < 0.05 by one-way *ANOVA* followed by Tukey’s *post hoc* test.

The expression of *TLR1-10* mRNA in the intestinal mucosa is shown in Figure 2. In the jejunal mucosa, all TLR types were expressed in NBW piglets at the same levels as LBW piglets (Figure 2A). However, LBW piglets had higher expression of *TLR2* in the ileal mucosa and *TLR8*, *TLR9*, and *TLR10* in colonic mucosa (*p* < 0.05; Figures 2B,C) but had lower expression of *TLR9* in the ileal mucosa as compared with NBW piglets (*p* < 0.05; Figure 2B).

In the jejunal mucosa of NBW-IgY piglets, the upregulated *TLR1*, *TLR2*, and *TLR6* expression (>3-fold; *p* < 0.05; Figure 2A) and the downregulated *TLR4* expression were seen as compared with NBW piglets (*p* < 0.05; Figure 2A). Likely, LBW-IgY piglets showed only upregulated *TLR1* and *TLR2* expression (>3-fold; *p* < 0.05; Figure 2A) in the jejunal mucosa.

In the ileal mucosa, GPD treatment downregulated only *TLR6* in NBW-IgY and LBW-IgY piglets compared to NBW (*p* < 0.05; Figure 2B). Treatment with GPD enhanced the TLR2 upregulation in the ileal mucosa of LBW piglets (*p* < 0.05; Figure 2B). However, *TLR1*, *TLR3*, *TLR4*, *TLR7*, *TLR8*, *TLR9*, and *TLR10* mRNA expressions were not modulated by GPD in the ileal mucosa of both LBW and NBW piglets (*p* > 0.05; Figure 2B).

The colonic mucosa of both NBW and LBW piglets treated with GPD demonstrated the elevated *TLR4*, *TLR5*, *TLR8*, and *TLR9* expression (>2-fold; *p* < 0.05; Figure 2C). The upregulated *TLR6*, *TLR7*, and *TLR10* were additionally observed in LBW-IgY piglets (*p* < 0.05; Figure 2C) as compared with NBW piglets. Nonetheless, the level of the increased *TLR8*, *TLR9*, and *TLR10* expression in LBW-IgY piglets was not different from that of LBW piglets (Figure 2C). Markedly, in the colonic mucosa, GPD treatment could not alter *TLR1*, *TLR2*, and *TLR3* expression in NBW and LBW piglets (*p* < 0.05; Figure 2C).

### 3.4 Effect of oral GPD on the growth performance and mRNA expression of cytokines in the intestinal mucosa of suckling piglets

**Figure 3:**
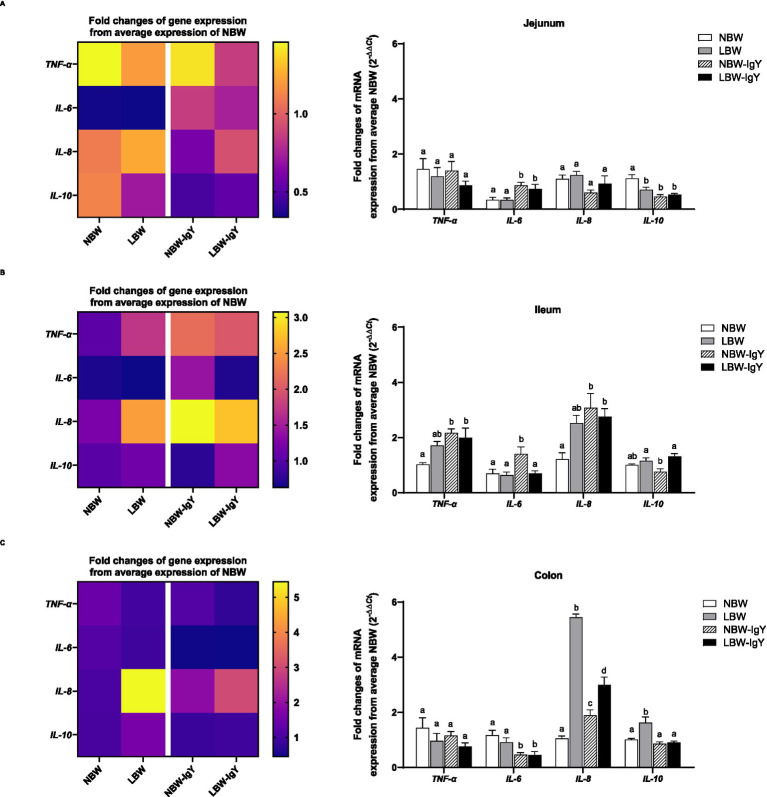
Effect of oral GPD on interleukin (*IL*)*-6*, −*8*, and −*10* and *tumor necrotic factor (TNF)-α* mRNA expression in **(A)** jejunal, **(B)** ileal, and **(C)** colonic mucosa of suckling piglets with normal birth weight (NBW) piglets vs. low birth weight (LBW) piglets. GPD was orally administered to NBW (NBW-IgY) and LBW (LBW-IgY) piglets at 6 and 10 h after birth. Total RNA was extracted from the intestinal mucosa of 7-day-old piglets. Expressions of related cytokines *IL-6*, *IL-8*, and *IL-10,* and *TNF-α* were determined by real-time RT-PCR as relative fold changes from the mean of NBW piglets using 2*^−∆∆Ct^* calculation. The heat map represents the mean, and the bars represent the mean ± SEM of individual gene expression conducted in a duplicated run (*n* = 5 piglets). The colors ranging from purple to yellow in the heat map for each gene reflect the fold changes of gene expression from lower to higher than NBW piglets. Bar graphs with different letters (a, b, c, or d) indicate significant differences among groups at *p* < 0.05 by one-way *ANOVA* followed by Tukey’s *post hoc* test.

The mRNA expressions of pro-inflammatory cytokines (*IL-6* and *TNF-α*), chemokines (*IL-8*), and anti-inflammatory cytokines (*IL-10*), but not interferons (*IFN-γ*), were detected in the intestinal mucosa of both NBW and LBW piglets. In all untreated intestinal mucosa, *TNF-α* and *IL-6* expression in NBW piglets did not differ from LBW piglets (Figures 3A–C). Compared with NBW piglets, LBW piglets revealed a significantly higher *IL-8* expression in the colonic mucosa (>5-fold; *p* < 0.05; Figure 3C) but not in the ileal or jejunal mucosa. Interestingly, the *IL-10* mRNA expression of LBW piglets was lower in the jejunal mucosa (*p* < 0.05; Figure 3A), but higher in the colonic mucosa than that of NBW piglets (*p* < 0.05; Figure 3C).

GPD elevated *TNF-α* expression only in the ileal tissue (*p* < 0.05; Figure 3A) but not in the jejunal or colonic mucosa of both NBW and LBW piglet groups (Figures 3B,C). Additionally, *IL-6* was upregulated in the jejunal mucosa of both NBW-IgY and LBW-IgY piglets (*p* < 0.05; Figure 3A), as well as in the ileal mucosa of NBW-IgY piglets (*p* < 0.05; Figure 3B). Unlikely, GPD downregulated *IL-6* expression in all colonic mucosa (*p* < 0.05; Figure 3C). In the ileal mucosa, GPD upregulated *IL-8* in both NBW-IgY and LBW-IgY piglets (*p* < 0.05; Figure 3B); however, the increased *IL-8* expression was not significantly different from untreated LBW piglets (Figure 3B). In the colonic mucosa, LBW piglets showed upregulated *IL-8* expression compared to NBW piglets (*p* < 0.05; Figure 3B). The GPD treatment could reverse the upregulated *IL-8* expression in LBW-IgY piglets to below that of LBW piglets (*p* < 0.05; Figure 3C), but the *IL-8* level in LBW-IgY piglets still remained higher than in NBW piglets (*p* < 0.05; Figure 3C). GPD increased *IL-8* expression in NBW-IgY piglets compared to NBW piglets (*p* < 0.05; Figure 3C); however, the level of increased *IL-8* expression was lower than in LBW or LBW-IgY piglets (*p* < 0.05; Figure 3C).

In the LBW piglets, *IL-10* expression was lower in duodenal mucosa but higher in colonic mucosa than NBW piglets (*p* < 0.05; Figures 3A,C). GPD could not reverse the downregulated *IL-10* expression in the duodenal mucosa; moreover, it suppressed *IL-10* in NBW-IgY (*p* < 0.05; Figure 3A). In contrast, in the colonic mucosa, GPD significantly reversed the upregulated *IL-10* expression caused by LBW-IgY piglets to the same level as NBW piglets (*p* < 0.05; Figure 3C). In the jejunal mucosa, the level of *IL-10* expression in NBW and LBW piglets was similar (*p* > 0.05; Figure 3B); however, GPD treatment downregulated the *IL-10* in NBW-IgY piglets (*p* < 0.05; Figure 3B).

### 3.5 Effect of oral GPD on the mRNA expression of neuropeptides related to inflammation in the intestinal mucosa of suckling piglets

**Figure 4:**
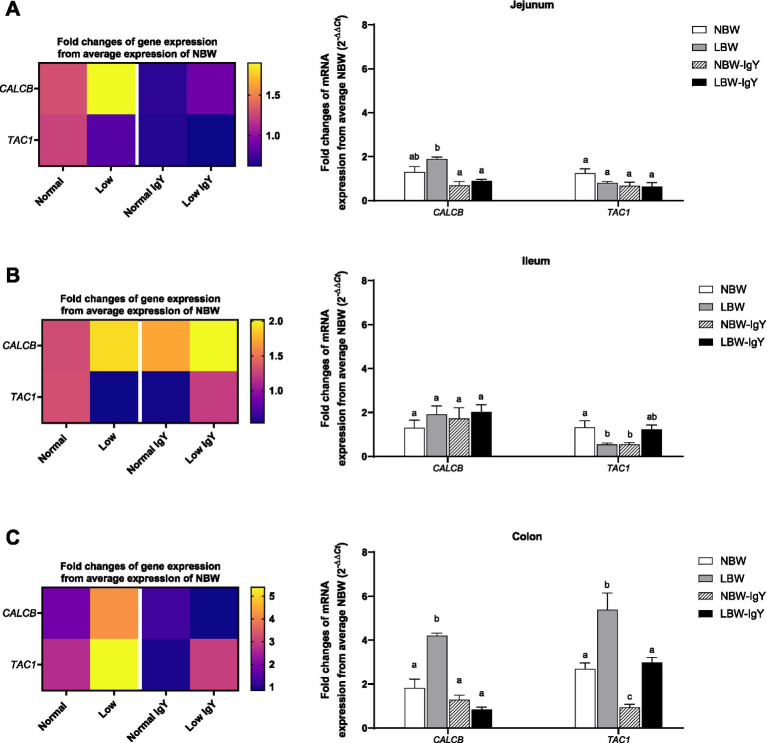
Effect of oral GPD on the mRNA expression of neuropeptides related to inflammation, calcitonin-related polypeptide beta (*CALCB*) and tachykinin precursor 1 (*TAC1*) mRNA expression in **(A)** jejunal, **(B)** ileal, and **(C)** colonic mucosa of suckling piglets with normal birth weight (NBW) piglets vs. low birth weight (LBW) piglets. GPD was orally administered to NBW (NBW-IgY) and LBW (LBW-IgY) piglets at 6 and 10 h after birth. Total RNA was extracted from the intestinal mucosa of 7-day-old piglets. Expression of *CALB* and *TAC1* was determined by real-time RT-PCR as relative fold changes from the mean of NBW piglets using 2*^−∆∆Ct^* calculation. The heat map represents the mean, and the bar represents the mean ± SEM of individual gene expression conducted in duplicated run (*n* = 5 piglets). The colors ranging from purple to yellow in the heat map for each gene reflect the fold changes of gene expression from lower to higher than NBW piglets. Bar graphs with different letters (a, b, or c) indicate significant differences among groups at *p* value *< 0.05* by one-way *ANOVA* followed by Tukey’s *post hoc* test.

The level of calcitonin-related polypeptide beta (*CALCB*) expression in jejunal and colonic mucosa in LBW piglets was higher than in other groups (>3-fold; *p* < 0.05; Figures 4A,C). Unlikely, LBW piglets had lower tachykinin precursor 1 (*TAC1*) expression in the ileal mucosa (*p* < 0.05; Figure 4B), but its *TAC1* expression in the colonic mucosa was higher than that of NBW piglets (2–4 fold; *p* < 0.05; Figure 4C).

GPD did not alter the *CALB* expression in any intestinal mucosa (Figure 4), whereas it downregulated *TAC1* expression in the ileal and colonic mucosa of NBW-IgY piglets (*p* < 0.05; Figures 4B,C). However, GPD reversed the upregulated *CALB* expression in the jejunal and colonic mucosa to the same level as NBW piglets (*p* > 0.05; Figures 4A,C). GPD also reversed the upregulated *TAC1* expression in the colonic mucosa of LBW-IgY piglets to the same level as in NBW piglets (*p* < 0.05; Figure 4C).

### 3.6 Effect of oral GPD on the mRNA and protein expression of tight junction proteins of suckling piglets

**Figure 5:**
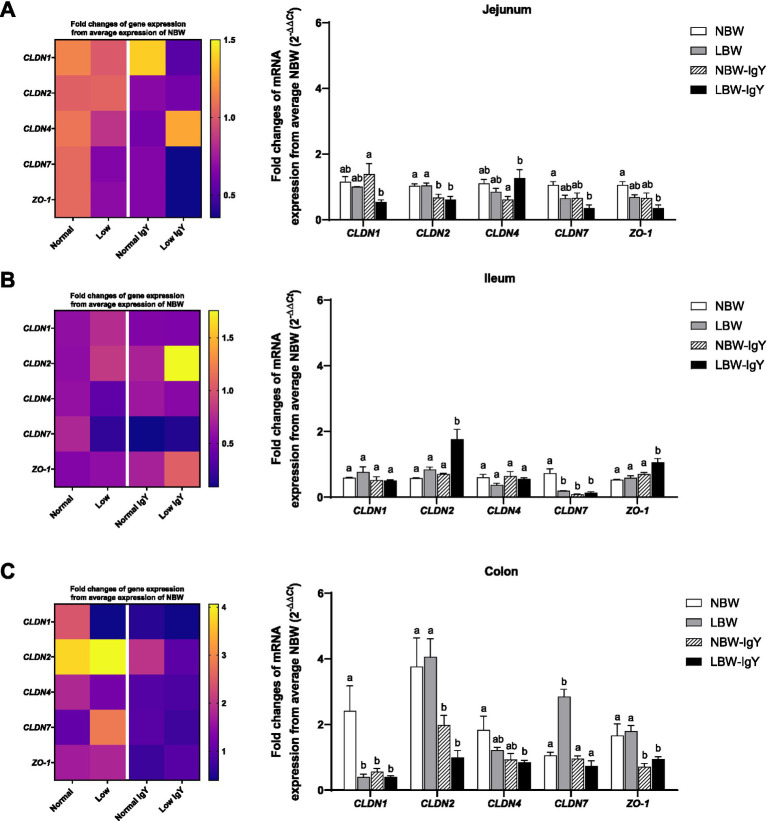
Effect of oral GPD on the mRNA expression of tight junction proteins in **(A)** jejunal, **(B)** ileal, and **(C)** colonic mucosa of suckling piglets with normal birth weight (NBW) piglets vs. low birth weight (LBW) piglets. GPD was orally administered to NBW (NBW-IgY) and LBW (LBW-IgY) piglets at 6 and 10 h after birth. Total RNA was extracted from the intestinal mucosa of 7-day-old piglets. Expressions of claudin, *CLDN1*, *CLDN2*, *CLDN4*, *CLDN7*, and zonula occludens-1 (*ZO-1*) were determined by real-time RT-PCR as relative fold changes from the mean of NBW piglets using 2*^−∆∆Ct^* calculation. The heat map represents the mean, and the bar represents the mean ± SEM of individual gene expression conducted in duplicated run (*n* = 5 piglets). The colors ranging from purple to yellow in the heat map for each gene reflect the fold changes of gene expression from lower to higher than NBW piglets. Bar graphs with different letters (a or b) indicate significant differences among groups at *p* < 0.05 by one-way *ANOVA* followed by Tukey’s *post hoc* test.

There was no difference in mRNA expression of all examined claudins (*CLDNs*), *CLDN1*, *CLDN2*, *CLDN4*, and *CLDN7*, and scaffold proteins Zonula Occludens-1 (*ZO-1*) in the jejunal mucosa of NBW and LBW piglets (Figure 5A). Unlikely, *CLDN7* expression in LBW piglets was lower in the ileal mucosa but higher in the colonic mucosa compared with those in NBW piglets (*p* < 0.05; Figures 5B,C, respectively). Additionally, the colonic mucosa of LBW piglets expressed a lower level of *CLDN1* than that of NBW piglets (*p* < 0.05; Figure 5C).

GPD supplementation suppressed *CLDN2* in the jejunal mucosa (*p* < 0.05; Figure 5A), *CLDN7* in the ileal mucosa (*p* < 0.05; Figure 5B), as well as *CLDN1* and *CLDN2* in the colonic mucosa of NBW-IgY piglets (*p* < 0.05; Figure 5C).

In the LBW-IgY piglets, *CLDN2* was downregulated in the jejunal and colonic mucosa, respectively (*p* < 0.05; Figures 5A,C). GPD increased *CLDN4* of LBW-IgY piglets in the jejunal mucosa compared with NBW-IgY piglets (*p* < 0.05; Figure 5A), but it did not significantly differ from NBW and LBW piglets (Figure 5A).

Interestingly, GPD downregulated *CLDN7* in the ileal mucosa (*p* < 0.05; Figure 5B) and downregulated *CLDN1* in the colonic mucosa of NBW piglets (*p* < 0.05; Figure 5C). Nonetheless, the downregulated *CLDN7* and *CLDN1* in LBW-IgY piglets could not be reversed by GPD treatment (*p* > 0.05; Figures 5B,C).

In addition to *CLDNs* expression, GPD upregulated *ZO-1* only in the ileal mucosa of LBW-IgY piglets (*p* < 0.05; Figure 5B) but downregulated *ZO-1* expression in the colonic mucosa of NBW-IgY and LBW-IgY piglets (*p* < 0.05; Figure 5C).

**Figure 6:**
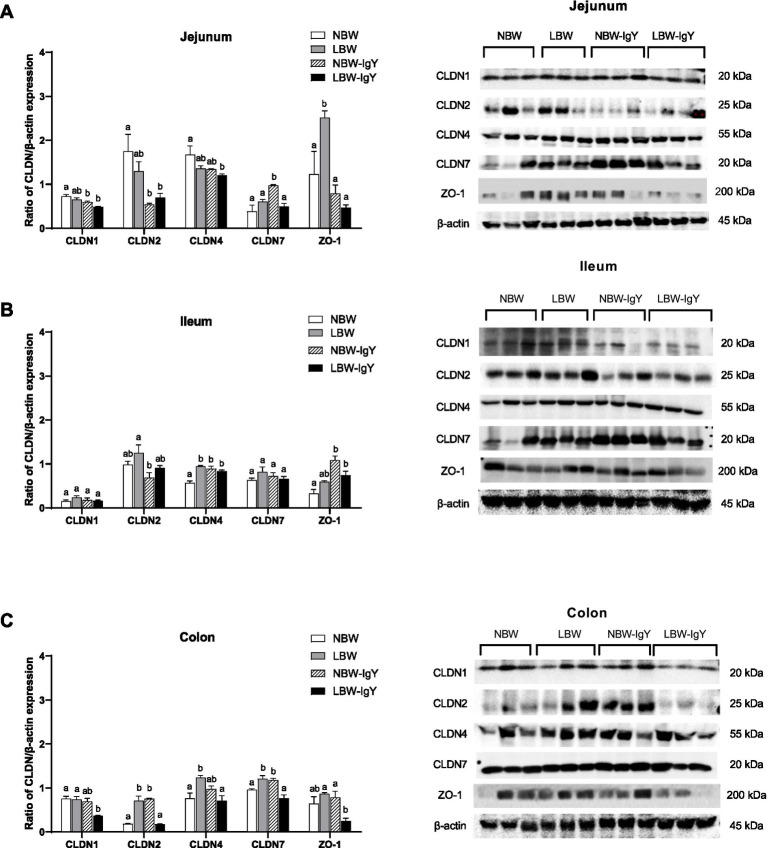
Effect of oral GPD on tight junction proteins expression in **(A)** jejunal, **(B)** ileal, and **(C)** colonic mucosa of suckling piglets with normal birth weight (NBW) piglets vs. low birth weight (LBW) piglets. GPD was orally administered to NBW (NBW-IgY) and LBW (LBW-IgY) piglets at 6 and 10 h after birth. Semi-quantitative Western blot analysis of tight junction proteins, claudins (CLDNs), CLDN1, CLDN2, CLDN4, and CLDN7, and zonula occludens-1 (ZO-1) were compared with β-actin. Representative protein bands of Western blot contributed to the quantitative analysis at 7 days after supplementation were presented in the right panel. Each bar represents the mean ± SEM of experiments conducted in duplicated run (*n* = 5 piglets). Bar graphs with different letters (a, b, or c) indicate significant differences among groups at *p* 0.05 by one-way *ANOVA* followed by Tukey’s *post hoc* test.

Relative protein expressions of the above CLDNs and ZO-1in the intestinal mucosa were further evaluated using Western blot analysis. LBW piglets upregulated ZO-1 in the jejunal mucosa (*p* < 0.05; Figure 6A), CLDN4 in the ileal mucosa (*p* < 0.05; Figure 6B), and CLDN2, CLDN4, and CLDN7 in the colonic mucosa (*p* < 0.05; Figure 6C) when compared with NBW piglets. Notably, GPD-treated LBW piglets reversed upregulation of ZO-1 in the jejunal mucosa and reversed the upregulation of CLDN2, CLDN4, and CLDN7 in the colonic mucosa to the same level as in the NBW piglets (*p* > 0.05; Figures 6A,C).

Nevertheless, GPD treatment in NBW piglets significantly upregulated CLDN7 in the jejunal mucosa (*p* < 0.05; Figure 6A). In addition, CLDN4 and ZO-1 in the ileal mucosa (*p* < 0.05; Figure 6B) and CLDN2 and CLDN7 in the colonic mucosa (*p* < 0.05; Figure 6C) were upregulated, but CLDN1 and CLDN2 were downregulated in the jejunal mucosa (*p* < 0.05; Figure 6A).

## 4 Discussion

Our study demonstrates that commercial oral IgY Globigen^®^ Pig Doser (EW Nutrition GmbH, Visbek, Germany; GPD) supplementation has a positive effect on growth in NBW piglets, significantly increasing the final weight at weaning. Although the GPD supplementation could not increase the final weight of LBW piglets to the same degree as NBW-IgY piglets, the final weight and ADG of LBW-IgY piglets were comparable to those of NBW piglets. These results indicate that the use of GPD supplementation can increase postpartum body weight and weaning weight of both NBW and LBW piglets.

Indeed, the control NBW group that was not treated also showed an increase in final weight and weight gain at 7 days. However, the results of the NBW-IgY group assessed at 24 days were higher than those of the untreated NBW group. Our study highlighted that GPD supplementation improves the local intestinal innate immunity, which is associated with the growth performance-enhancing potential of GPD in pre-weaned piglets. Globigen^®^ Jump Start (EW Nutrition GmbH, Visbek, Germany) supplementation provides passive immunity against multiple common pathogens challenged piglets during the first weeks of life, that is, *E. coli* (ETEC) F4 and F18, *E. coli* K88, *Clostridium perfringens*, rotavirus, and *S. typhimurium* (17, 24), but not a direct effect on growth performance.

Pathogenic *E. coli* strains, the most common pathogens on the first day, particularly ETEC, utilize fimbriae (also known as pili) to adhere to the intestinal mucosa of piglets. This adherence is crucial for colonization and subsequent infection (25). Once attached, these bacteria produce enterotoxins, such as heat-labile enterotoxin (LT) and heat-stable enterotoxin (ST), which disrupt the normal fluid balance in the intestines, leading to diarrhea (26).

Possibly, administering GPD blocked bacterial adhesion to intestinal mucus, reduced colonic bacterial loads, and stabilized inflammatory cytokine levels, thereby improving gut health and potentially supporting better growth performance (18). Even though the commercial oral GPD improved growth performance in LBW-IgY to NBW piglets, the results were not significantly different from LBW piglets.

In addition to IgY against specific pathogens, as in Globigen^®^ Jump Start (EW Nutrition GmbH, Visbek, Germany), Globigen^®^ Pig Doser (EW Nutrition GmbH, Visbek, Germany) contains other active gradients, including soybean oil, vitamins A, D3, and E, and probiotics *Enterococcus faecium* (24), aiming to reduce diarrhea and intestinal inflammation in piglets. The anti-inflammatory effect of commercial oral GPD supplementation may protect physiological processes in terms of energy conservation against fever and anorexia, thereby causing a reduction in pig performance. Interestingly, soybean oil not only facilitates the delivery of IgY but also provides additional nutritional benefits (27). It appears that the potential immunity effects of oral IgY supplementation depend on proper epithelial integrity. Since LBW piglets were indicated to have lower small intestine development and function than NBW piglets (28), the response of LBW-IgY piglets was not comparable to that of NBW-IgY piglets. However, vitamin A, D, and E are essential vitamins for growth and development, promoting the expression of barrier TJs protein, such as *Cldn-1* and *Occludin1* expression in jejunum and ileum (29, 30). In addition, the expressions of inhibitory cytokines, such as *IL-4*, *IL-5*, and *IL-10*, are upregulated, while the expression of pro-inflammatory cytokines, including *IL-1β*, is downregulated by these essential vitamins (29, 30)*. E. faecium* is a beneficial bacterium that promotes intestinal health (31). It raised the possibility that GPD reduces gastrointestinal infections during supplementation and may have a long-term effect promoting the cellular target of mucosal immunity in all post-weaned piglets.

The cellular target of GPD in distinct intestinal tissues was of interest since each intestinal site has a specific gut health responsibility. The jejunum is the primary region to absorb most nutrients, the ileum, consisting of Peyer’s patch, exerts immunity and barrier function, and the colon is mainly involved in water reabsorption (32) is colonized by a large microbial population (59). Our current results indicate that the expression of tight junction genes and proteins, as well as the expression of genes related to mucosal barrier and immunity, in the jejunal, ileal, and colonic mucosa was most considered and found to be modulated by GPD supplementation to varying degrees.

Porcine β-defensins (PBDs) are antimicrobial peptides (AMP) that play an important role in the innate immune response in the intestine (33, 34). In the present study, using the ileal mucosa of LBW piglets, GPD supplementation was found to upregulate *PBD2* and *PBD4* without affecting the low level of *PBD1* expression. The low constitutive *PBD1* expression may be of less significance since PBD2 and PBD3, rather than PBD1, are inducible subtypes in response to invasive microorganisms (35). As the pig PBD2 or human BD4 has antimicrobial activity against *S. typhimurium*, *E. coli*, *C. perfringens*, or *Pseudomonas aeruginosa* (36, 37). Our data indicate that GPD supplementation increases the expression of PBDs, notably in the intestinal mucosa of LBW piglets, where the gut immune system may be underdeveloped or impaired. Improving PBD expression through GPD supplementation may be especially effective in providing antibacterial protection.

The induction of PBD synthesis by both microbial and chemical stimuli is mediated by hormonal receptors, including the epidermal growth factor receptor (EFGR) (38–40). Chicken egg powder-based immunoglobulin, Globigen^®^ Pig Doser (EW Nutrition GmbH, Visbek, Germany), contains specific antibodies raised against pathogens in piglets, that is, enterotoxigenic *E. coli* (ETEC) (24). Hyperimmunized IgY and critical proteins in egg yolks may enhance intestinal immune system development, notably innate immune system PRRs and signaling system to trigger all epithelial pathogen responses.

TLRs are one of the major PRRs that are expressed by immune and non-immune cells, including intestinal epithelial cells (41). All *TLR1-10* subtypes were identified in the intestinal tissues used in the present study. These TLRs play significant roles in promoting both adaptive and regulatory immunity. For example, TLR8 and TLR9 are responsible for the production of antiviral molecules such as type I interferons (60), whereas TLR2, TLR4, and TLR6 stimulate an inflammatory response against pathogenic bacteria and probiotics (42). Moreover, TLR2 and TLR4 simultaneously stimulate the production of IL-10, an anti-inflammatory cytokine, which inhibits the overresponse from inflammation (61).

In the present results, the *TLR1*, *TLR2*, and *TLR6* in NBW-IgY and LBW-IgY piglets’ jejunum, as well as the TLR2 in LBW-IgY piglets’ ileum, were upregulated. Even though the evidence does not seem significant in the small intestinal tissues, which have the principal function in nutrient absorption, the upregulated *TLRs* may help the small intestine mucosa respond to microorganisms during food transit in the lumen. In colonic mucosa, the upregulation of several *TLRs*, *TLR4*, *TLR5*, *TLR8*, and *TLR9* in NBW-IgY piglets and *TLR4*, *TLR5*, and *TLR10* in LBW-IgY piglets indicates that GPD may help promote microbial recognition at the colonic mucosa. Conversely, the upregulation of *TLR2*, *TLR8*, *TLR9*, and *TLR10*, especially in the colonic mucosa of LBW piglets, may cause undesired effects. In the stress weaning piglets, pathogenic bacteria, including *E. coli*, a diarrhea-causing bacterium directly binds cell-surface molecules such as gangliosides GM1 (43), inducing the cellular response including the expression of many genes associated with intestinal mucosal immunity, that is, *TLRs*, pro-inflammatory cytokines or tight junction proteins (44). High levels of colonic *TLRs* expression may cause the over-response of LBW piglets to all microorganisms, including residential microflora in the colon resulting in chronic inflammation. The evidence may be associated with poor growth performance in LBW piglets.

Cytokines play crucial roles in the pig intestinal system by mediating immune responses, regulating inflammation, and affecting overall gut health. In the present study, the expression of relevant cytokines was detected except for *IFN-γ*. The primer sets used in this study were tested and reported in our previous study (22). However, the level of *IFN-γ*. expression in intestinal mucosa was undetectable. Thus, the result of IFN-*γ* expression was not demonstrated in the figure. The expression level of cytokine genes *TNF-α* and *IL-6* by the intestinal mucosa of LBW piglets was similar to that of NBW piglets. In contrast, the expression of *IL-8* in in the colonic mucosa of LBW piglets was generally greater than in NBW piglets. The increased TNF-α, IL-1β, and IL-8 in the hindgut mucosa of LBW piglets has been previously reported (45). IL-8 plays a key role in the early inflammatory response by activating monocytes and recruiting neutrophils to the site of inflammation (46). Therefore, high expression of *IL-8* in the colonic mucosa of LBW piglets indicated a proneness to low-grade inflammation (45). However, upregulated TNF-*α* and IL-6 coincided with downregulated IL-10 contributes to intestinal inflammation and barrier dysfunction (47). Perhaps, our finding of the upregulation of inhibitory cytokines *IL-10* in the colonic mucosa may help in managing the inflammation in LBW piglets. Even though the downregulated *IL-10* was found in the jejunal mucosa of LBW piglets, it did not coincide with the upregulated pro-inflammatory cytokines. Thereby, gastrointestinal disorders or enteritis were not shown in all LBW piglets during the suckling period. Nonetheless, the long-term effects of the changes in cytokine levels need to be monitored.

Our findings reveal that GPD treatment altered cytokine profiles relevant to TLR expression in different segments of the intestinal mucosa. GPD increased *IL-6* in the jejunal mucosa and *TNF-α* and *IL-8* in the ileal mucosa, but decreased *IL-10*, especially in the jejunal and ileal mucosa of NBW piglets and LBW piglets. As mentioned earlier, based on cytokines levels, GPD treatment indicates a shift toward a more pro-inflammatory environment, which could improve pathogen clearance but also increase the risk of inflammation-related tissue damage. Coincidentally, GPD-upregulated *TLRs* improved colonic mucosa responsiveness to recognizing pathogens in all piglets. Meanwhile, GPD reversed *IL-8* overexpression in colonic mucosa of LBW piglets, which may improve intestinal immune response by preventing overactive immune response, causing intestinal mucosa damage.

Enteric nervous system neurochemicals CGRP and SP govern secretomotor reflex, motility, and secretory response to modulate bacterial toxin sensing and adherence (48, 49). Early weaning stress causes as long-term reduction in gut barrier function along with increased secretomotor activity. The suggested mechanisms involved corticotropin-releasing factor (CRF) signaling pathways and inflammatory cytokine responses to stimulate cholinergic secretory activities (50). However, the increase in cholinergic neurons did not completely explain the increased neurosecretory diarrhea in early weaned piglets (50). Instead, high levels of *TAC1* and *CALCB*, which encode SP and CGRP, are related to several pathologic states, including GI inflammation and nerve damage (51). Our results found the elevated *TAC1* and *CALCB* only in the LBW piglets’ colonic mucosa, which was correlated with a greater level of IL-8. Interestingly, these elevated neuropeptides and *IL-8* expression levels were reversed by the GPD supplementation, indicating that GPD may improve gastrointestinal health in LBW piglets by inhibiting the adherence of luminal pathogens to the intestinal mucosa.

TJs are multiprotein complexes crucial for intestinal barrier function, regulating the paracellular permeability of ions, water, and molecules (52). The present study focused on alterations of barrier builder (CLDN1, CLDN4, CLDN7, and ZO-1) and pore forming (CLDN2) TJ mRNA and protein expression in response to birth weight or GPD. Both NBW and LBW piglets showed different TJ mRNA and protein expression in the intestinal mucosa; however, only the increased CLDN7 protein was associated with gene expression in the colonic mucosa. However, the regulation of TJs protein expression should be more thoroughly examined because it exploits functions in gut integrity.

In LBW piglets, the upregulated barrier builder TJs, ZO-1 in the jejunal mucosa, and CLDN4 in the ileal mucosa were found. Additionally, CLDN2, CLDN4, and CLDN7 were upregulated in the colonic mucosa. The increased barrier TJs in the early stage of life in LBW piglets from the “open state” to “close state” or leaky to tight epithelia may be disadvantageous to paracellular permeability for growth factors and immunoglobulins presented in colostrum/milk to be transported (53).

In contrast, overexpression of pore-forming TJs, CLDN2, in the colonic mucosa where the pathogens are colonized may be harmful (54). These findings indicate an adaptive response in LBW piglets to maintain intestinal barrier integrity; however, TJs that mediate epithelial tightening may be overexpressed or imbalanced. GPD treatment modulated TJ expressions in LBW piglets, especially the increased ZO-1 in the jejunal mucosa to levels comparable to NBW piglets. Although the IgY decreased barrier TJs, CLDN1, CLDN4, and ZO-1 and pore-forming TJ, CLDN2, in LBW piglets’ colon, it should be considered as a benefit because the growth retardation of LBW piglets were reversed to normal (Figure 6C; Table 2).

In NBW piglets, GPD promotes growth performance by increasing the characteristics of tight epithelia through upregulation of CLDN4, CLDN7, and ZO-1, while downregulating CLDN2 in jejunal and ileal regions. In addition to maintaining gut integrity, upregulated ZO-1 is involved in cell signaling pathways that regulate cell proliferation, differentiation, survival, and repair. Dietary supplement mitigates the effects of enterotoxigenic *E. coli* (ETEC) infections by modulation of ileal microbiota involving the increase of ZO-1 expression (55). The increased ZO-1 was also widely distributed under conditions of faster cell turnover/proliferation (56).

Moreover, another study found that high dietary calcium and microbial phytase intake enhanced the expression of CLDN4 and CLDN7, promoting paracellular calcium absorption, which is vital for nutrient absorption and gut health (57). IgY treatment downregulated the cation-pore-forming TJ, CLDN2 expression; however, CLDN2 is often associated with inflammation and barrier dysfunction in response to inflammatory signals (58). Perhaps, like other diets, GPD may promote post-birth bacterial colonization by influencing the expression of TJs or directly enhance gut maturation, promoting intestinal health in suckling piglets. Nevertheless, the GPD effect, by improving intestinal morphology and barrier function, promoting beneficial gut microbiota, and improving outcomes in the GIT of piglets, needs to be further confirmed.

## 5 Conclusion

Application of commercial IgY-based supplement Globigen^®^ Pig Doser (EW Nutrition GmbH, Visbek, Germany) demonstrates a positive effect on growth performance in suckling piglets, whether the piglet has a low or normal birth weight. GPD supplementation also enhances intestinal mucosal immunity by modulating the expression of the innate immune system, that is, TLRs, cytokines, and tight junction genes, particularly in LBW piglets, which have a distorted development from NBW piglets. The study’s findings may serve as an informative tool for interested parties and provide guidelines for management to improve gut health, particularly in LBW piglets, thereby preventing pre-weaning losses.

## Data Availability

The datasets presented in this study can be found in online repositories. The names of the repository/repositories and accession number(s) can be found in the article/Supplementary material.
