# Supplementary figures and images for: Cellular effects of oral egg yolk immunoglobulin-based supplementation at birth on promoting growth and strengthening intestinal mucosal innate immunity in pre-weaned piglets
Source: Front Vet Sci. 2025 Jul 16;12:1458279. doi: 10.3389/fvets.2025.1458279 (PMC12309497; doi:10.3389/fvets.2025.1458279)

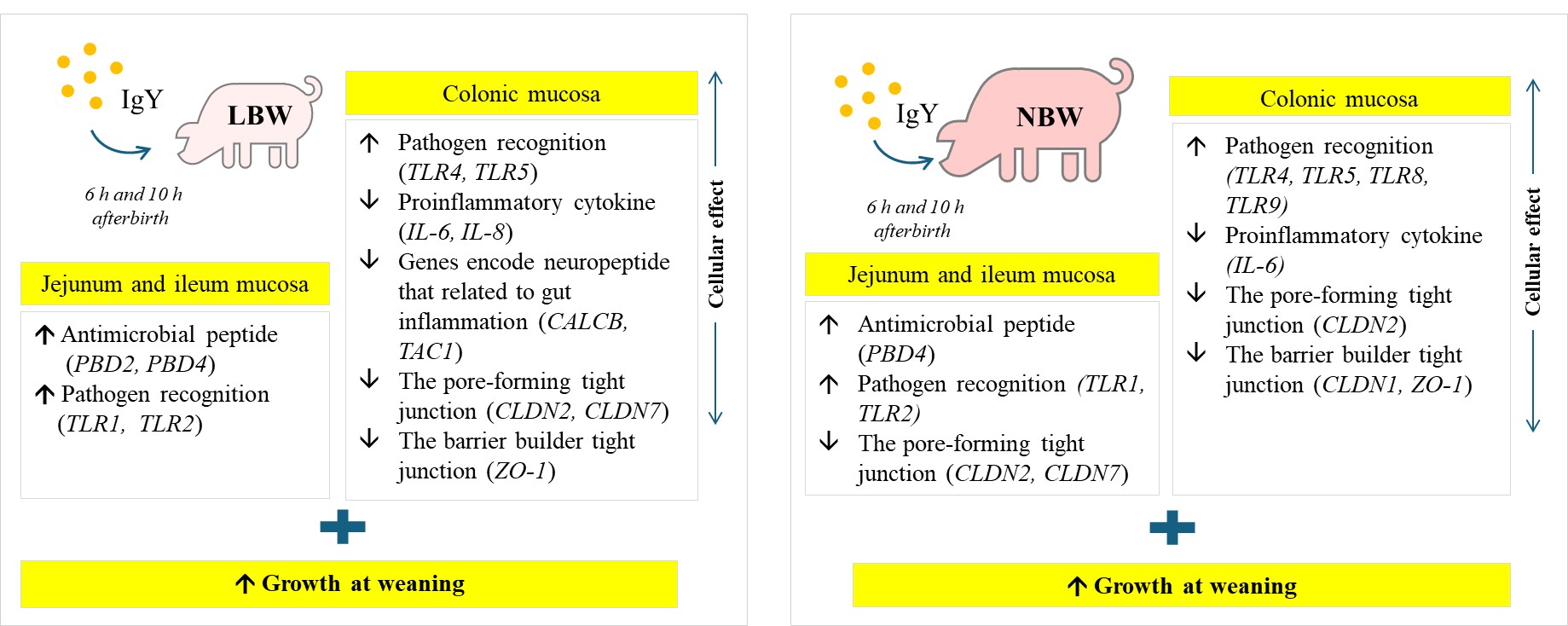

Supplement: Supplementary file 1 [file Image_1.jpeg]
